# Malignant Solitary Fibrous Tumor of the Breast: A Case Report and Selected Review of the Literature

**DOI:** 10.70352/scrj.cr.26-0640

**Published:** 2026-11-07

**Authors:** Kazunoshin Tachibana, Yuichiroh Kiko, Akihiko Ozaki, Maiko Okano, Masaru Noda, Nobuhiro Hoshi, Sadahiko Abe, Kako Minami, Mari Hashimoto, Taeka Terui, Yuko Hashimoto, Tohru Ohtake

**Affiliations:** 1 Department of Breast Surgery, Fukushima Medical University, Fukushima, Fukushima, Japan; 2 Department of Diagnostic Pathology, Fukushima Medical University, Fukushima, Fukushima, Japan; 3 Department of Breast Surgery, Jyoban Hospital of Tokiwa Foundation, Iwaki, Fukushima, Japan

**Keywords:** solitary fibrous tumor, breast, malignant tumor, signal transducer and activator of transcription 6 (STAT6)

## Abstract

**INTRODUCTION:**

Solitary fibrous tumor (SFT) is a rare mesenchymal neoplasm that may arise at various anatomical sites. Breast SFT is uncommon, and malignant SFT of the breast is exceptionally rare and can be difficult to distinguish from other spindle cell tumors.

**CASE PRESENTATION:**

A 67-year-old woman presented with a rapidly enlarging mass in the right breast. Imaging demonstrated a well-circumscribed, hypervascular tumor that increased from approximately 10 to 16 cm within 1 month. Core needle biopsy suggested a stromal sarcoma or a malignant phyllodes tumor, and a total mastectomy was performed. Histopathological examination showed a patternless spindle cell proliferation with staghorn-like vessels, frequent mitoses, and necrosis. The tumor cells showed diffuse nuclear signal transducer and activator of transcription 6 (STAT6) positivity and were positive for cluster of differentiation (CD) 34, CD99, β-catenin, B-cell lymphoma 2 (BCL2), and smooth muscle actin. Malignant SFT was diagnosed. The patient remains disease-free 5 years after surgery without adjuvant therapy.

**CONCLUSIONS:**

A malignant SFT should be considered in the differential diagnosis of rapidly growing breast spindle cell tumors. Complete surgical excision with negative margins and STAT6-based histopathological confirmation can support an accurate diagnosis and favorable long-term outcomes.

## Abbreviations

BCL2
: B-cell lymphoma 2
BI-RADS
: Breast Imaging Reporting and Data System
CD
: cluster of differentiation
CE-CT
: contrast-enhanced computed tomography
EGR1
: early growth response 1
EMA
: epithelial membrane antigen
ER
: estrogen receptor
FDG-PET/CT
: ^18^F-fluoro-2-deoxy-D-glucose positron emission tomography/computed tomography
HER2
: human epidermal growth factor receptor 2
Ki-67
: marker of proliferation Ki-67
PgR
: progesterone receptor
p63
: tumor protein 63
SFT
: solitary fibrous tumor
SMA
: smooth muscle actin
STAT6
: signal transducer and activator of transcription 6
SUVmax
: maximum standardized uptake value

## INTRODUCTION

SFT is an uncommon mesenchymal neoplasm that can be found at any location, with approximately 40% of cases occurring within subcutaneous tissues.^1)^ The entity was initially described by Klemperer and Rabin in 1931 as a pleural tumor.^2)^ While it may arise at various sites such as the head and neck, thoracic wall, mediastinum, abdominal cavity, spinal cord, lungs, thyroid, liver, gastrointestinal tract, adrenal glands, and kidneys, its occurrence within the breast is exceedingly rare, with only 33 cases documented in the English-language literature,^3)^ and malignant SFT of the breast is an exceptionally uncommon entity with only a few cases documented in the English-language literature.^4,5)^

Given its rarity and the overlapping features with other spindle cell neoplasms, such as malignant phyllodes tumors or sarcomas, the diagnosis and management of malignant SFT of the breast remain challenging. Furthermore, treatment strategies for rapidly enlarging or histologically malignant mesenchymal tumors of the breast are not well established due to the paucity of reported cases. Herein, we report a case of malignant SFT of the breast presenting with rapid growth and review the literature to highlight the diagnostic pitfalls and therapeutic considerations associated with this rare entity.

## CASE PRESENTATION

A 67-year-old woman presented to a local clinic after noticing a palpable mass, approximately 9 cm in diameter, in her right breast. She was subsequently referred to Fukushima Medical University Hospital for further evaluation. Her medical history was unremarkable, and she had no family history of breast malignancy. Clinical examination revealed a firm, elastic, and smooth-surfaced mass measuring 10 cm in the upper outer quadrant of the right breast. The overlying skin appeared erythematous without nipple discharge, and no axillary lymphadenopathy was noted.

Mammography revealed a well-circumscribed, high-density mass (**Fig. 1**). Ultrasonography demonstrated a smooth-marginated, hypoechoic mass, classified as BI-RADS Category 4C. Core needle biopsy raised the suspicion of a stromal sarcoma or a malignant phyllodes tumor. Immunohistochemical analysis revealed that the tumor was negative for ER, PgR, and HER2, but positive for SMA, CD34, and BCL2. The tumor cells were also negative for the broad-spectrum cytokeratin AE1/AE3, desmin, h-caldesmon, and S-100 protein, and the Ki-67 labeling index was 20%. Spindle cell metaplastic carcinoma was included in the preoperative differential diagnosis but was considered unlikely because no carcinomatous or in situ epithelial component was identified in any of the core biopsy specimens, and the spindle cells were negative for AE1/AE3 while diffusely expressing CD34. The absence of desmin and h-caldesmon argued against a smooth muscle tumor, and S-100 negativity against a peripheral nerve sheath tumor. STAT6 immunostaining was not performed on the biopsy specimen. These findings raised suspicion for a malignant phyllodes tumor.

**Fig. 1:**
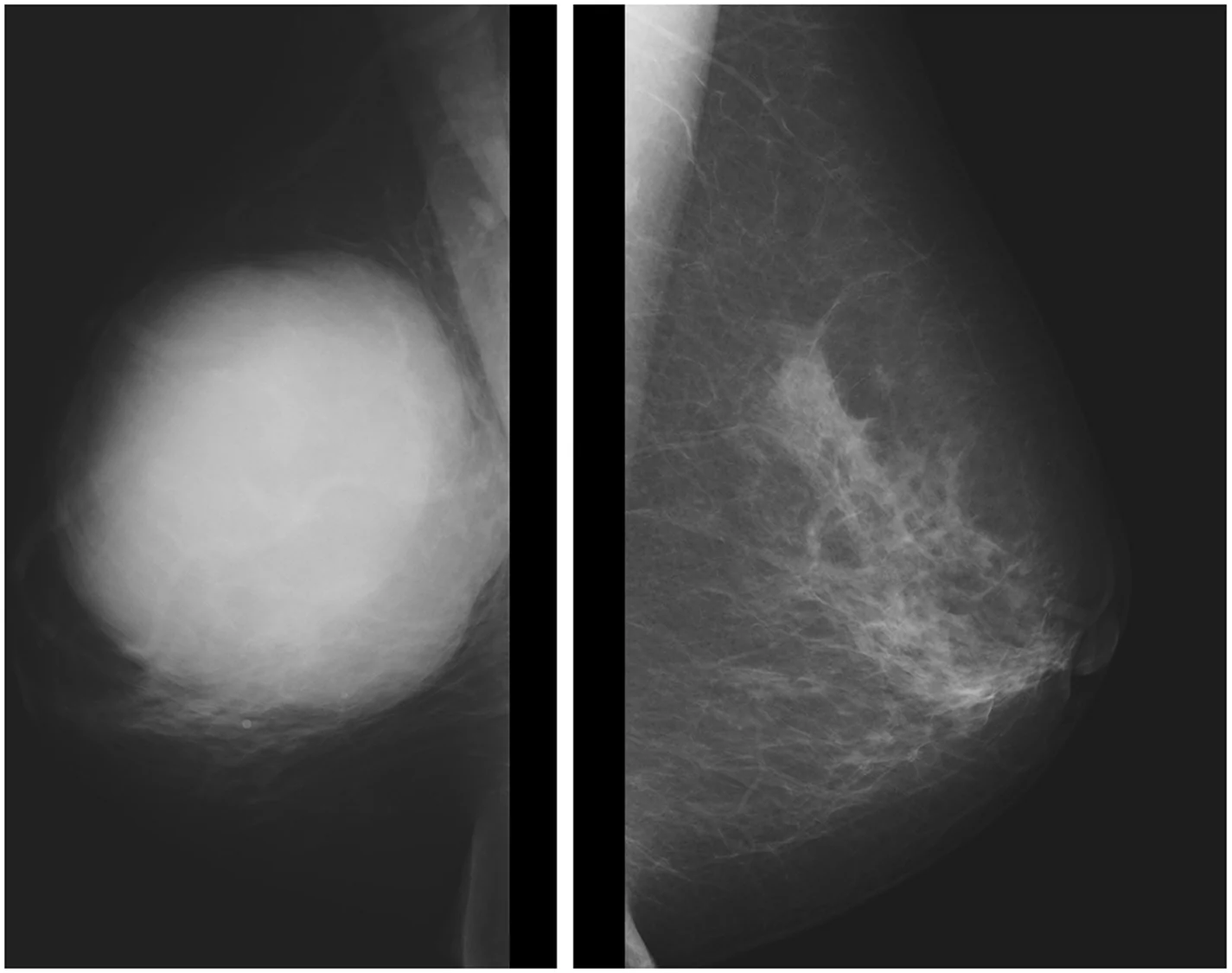
Mammographic appearance of the breast tumor. Mammography showed an oval, well-circumscribed, high-density mass in the right breast.

CE-CT demonstrated a heterogeneously enhancing solid mass with a maximum diameter of 11 cm (**Fig. 2A**). FDG-PET/CT revealed high peripheral uptake with an SUVmax of 7.4 (**Fig. 2B**). MRI showed low signal intensity on T1-weighted images and high signal intensity on fat-suppressed T2-weighted images (**Fig. 2C**, **2D**). Prominent peritumoral vascular structures were evident, and no distant metastases were identified. The CE-CT, FDG-PET/CT, and MR images shown in **Fig. 2** were all obtained in the axial plane at the level of the maximum transverse diameter of the tumor; at this level, the tracer uptake formed a ring-shaped peripheral rim with a photopenic center (**Fig. 2B**).

**Fig. 2:**
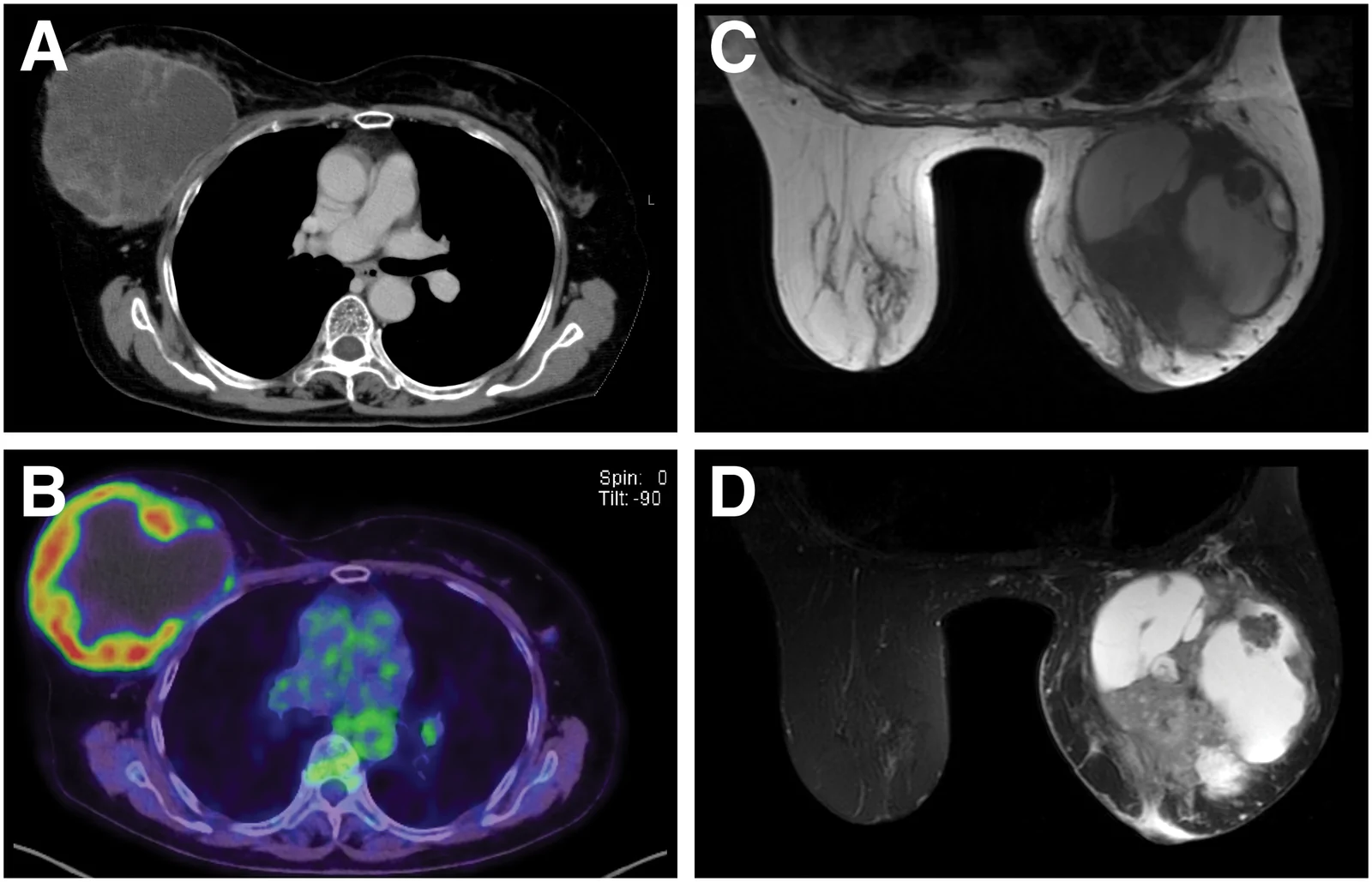
CT, PET, and MRI. (**A**) CE-CT showed a heterogeneously enhancing solid mass. (**B**) Fluorodeoxyglucose PET/CT showed high uptake in the tumor periphery, with a SUVmax of 7.4. (**C**) MRI showed low signal intensity on a T1-weighted image. (**D**) MRI showed high signal intensity on a fat-suppressed T2-weighted image. All images are displayed in the axial plane at the level of the maximum transverse diameter of the tumor, corresponding to the plane of the cut surface shown in **Fig. 4D**. In (**B**), the ring-shaped peripheral uptake corresponds to the viable peripheral portion of the tumor, and the photopenic center corresponds to the central hemorrhagic and necrotic area. CE-CT, contrast-enhanced computed tomography; SUVmax, maximum standardized uptake value

The mass demonstrated progressive enlargement, reaching approximately 16 cm by the time of surgery (**Fig. 3**). A total right mastectomy was performed due to clinical and pathological suspicion of a malignant phyllodes tumor (**Fig. 4**). Histopathological examination revealed spindle-shaped cells arranged in a patternless architecture surrounding thin-walled, branching staghorn-like vessels, with frequent mitotic figures (32 per 50 high-power fields, corresponding to approximately 6.4 per 10 high-power fields) and extensive necrosis in the central portion of the tumor (**Fig. 5**). No epithelial components were observed. The plane of the cut surface of the mastectomy specimen (**Fig. 4D**) corresponded to that of the axial FDG-PET/CT image (**Fig. 2B**): the ring-shaped peripheral uptake corresponded to the solid, viable peripheral portion of the tumor, whereas the photopenic center corresponded to the central hemorrhagic and necrotic area, which was confirmed histologically (**Fig. 5A**). Immunohistochemically, the tumor cells exhibited diffuse and strong nuclear positivity for STAT6, along with positivity for CD34, CD99, β-catenin, BCL2, and SMA. They were negative for desmin, AE1/AE3, EMA, and S-100. The Ki-67 index was elevated at 37% (**Fig. 5**). The surgical margins were tumor-free, and the final pathological diagnosis was malignant SFT. The patient did not receive adjuvant therapy and has remained disease-free for 5 years postoperatively.

**Fig. 3:**
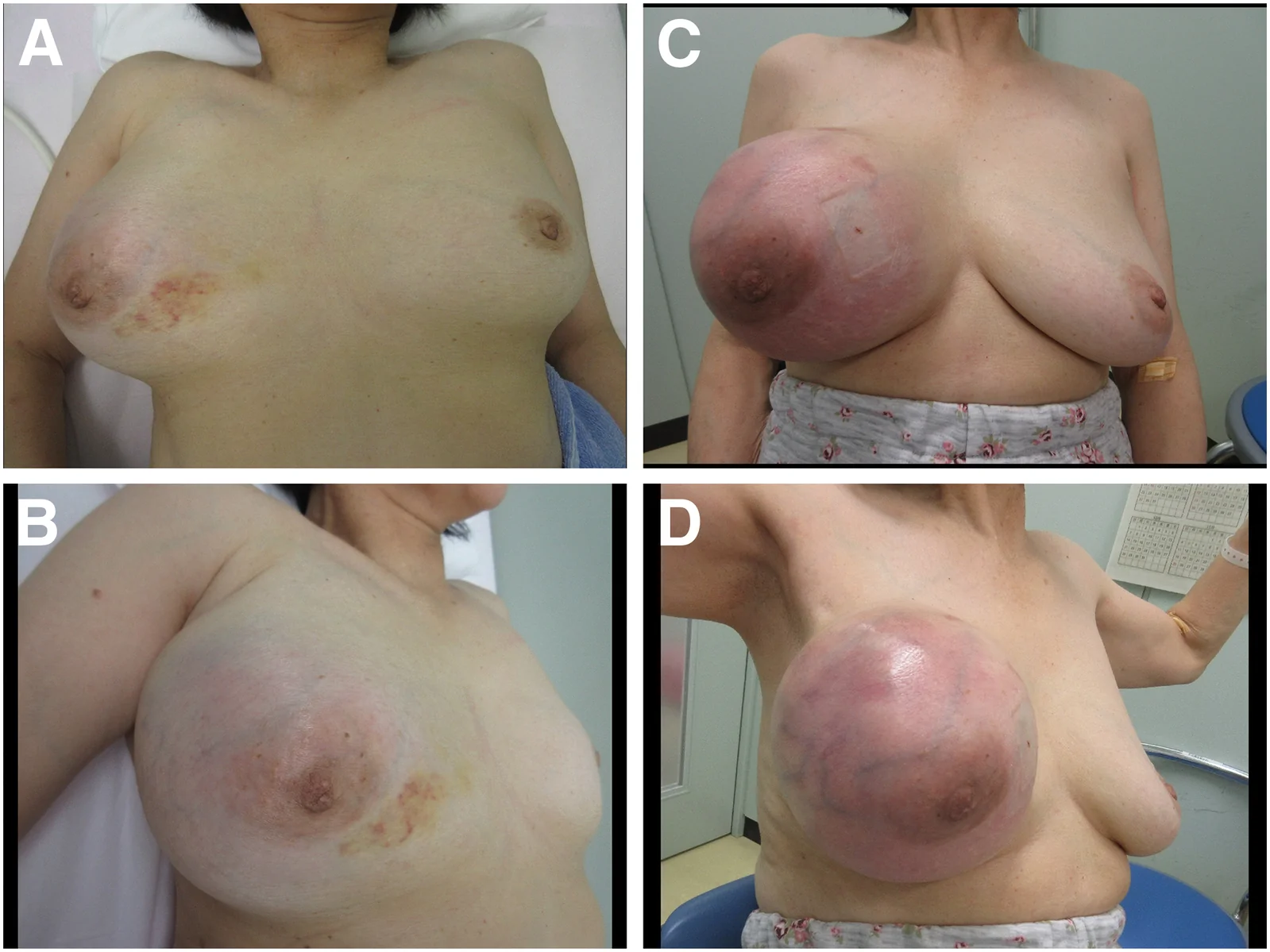
Rapid growth of the right breast tumor. (**A** and **B**) The tumor measured 10 cm at the initial visit. (**C** and **D**) The tumor had enlarged to 16 cm 1 month after the initial visit.

**Fig. 4:**
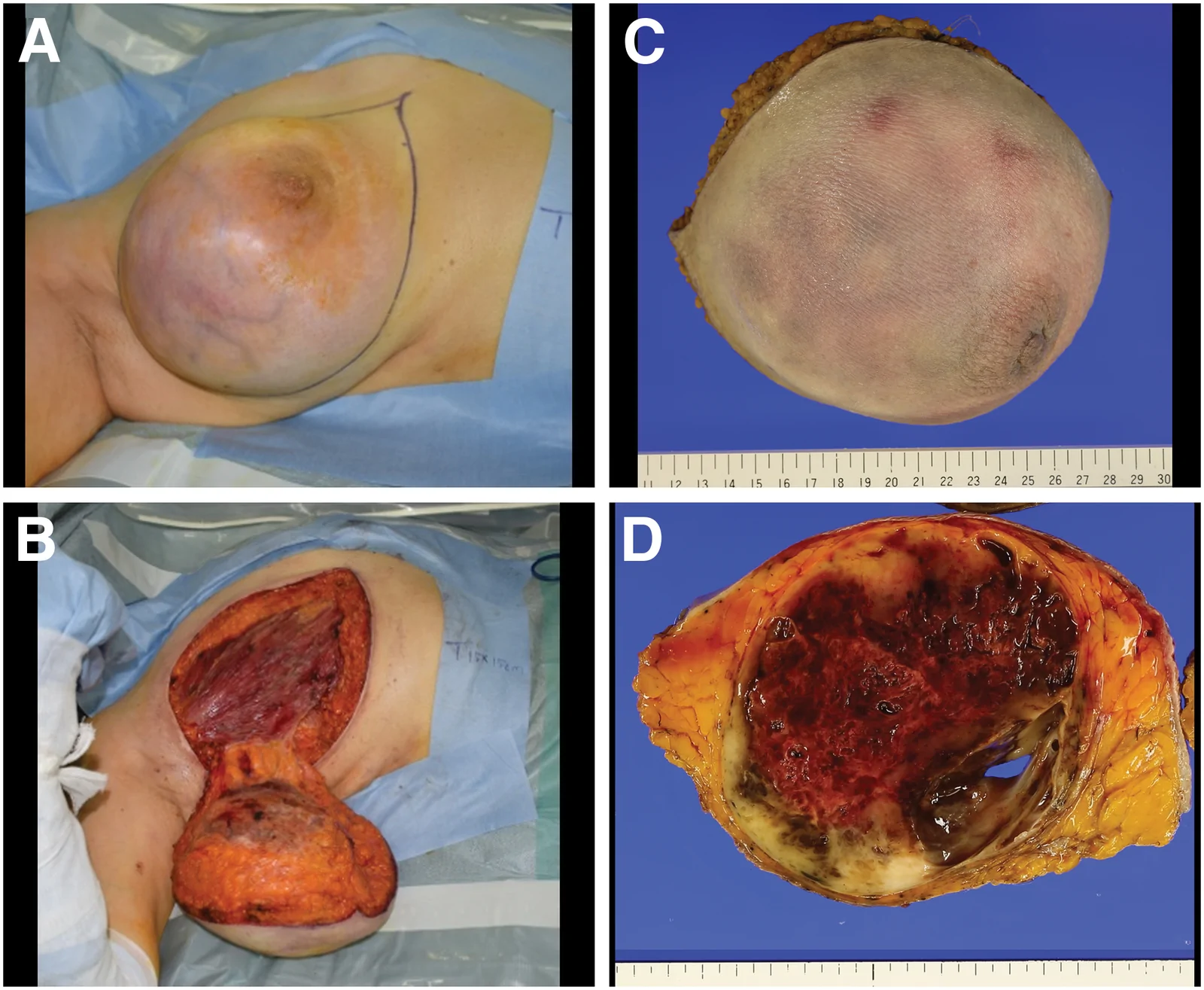
Mastectomy and gross pathological findings. (**A** and **B**) Right total mastectomy. (**C**) Gross appearance of the mastectomy specimen. (**D**) The cut surface of the specimen, sectioned in the axial plane corresponding to **Fig. 2**, showed central hemorrhage and necrosis surrounded by a rim of solid, viable tumor.

**Fig. 5:**
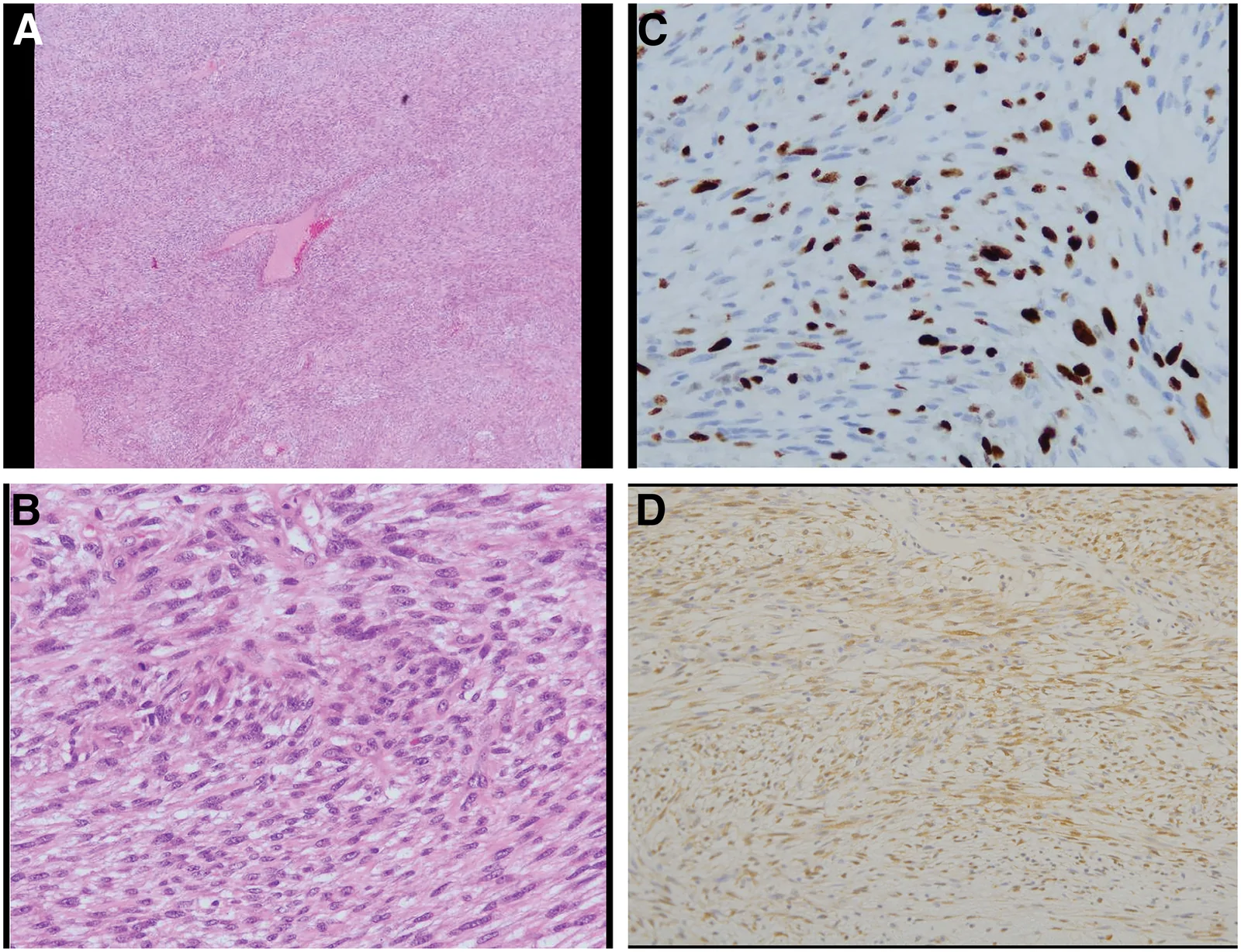
Histopathological and immunohistochemical findings. (**A**) Hematoxylin and eosin staining (original magnification, ×100) showed spindle-shaped cells in a patternless architecture around thin-walled, staghorn-like vessels, with frequent mitoses and necrosis. (**B**) Hematoxylin and eosin staining (original magnification, ×400) showed stromal overgrowth and frequent mitoses. (**C**) Immunohistochemical staining for CD34 (original magnification, ×400). (**D**) Immunohistochemical staining for STAT6 (original magnification, ×400). STAT6, signal transducer and activator of transcription 6

## DISCUSSION

This case highlights the diagnostic difficulty of malignant SFT of the breast, a rare entity that can mimic other rapidly enlarging mesenchymal neoplasms. Preoperative imaging and core needle biopsy suggested a malignant phyllodes tumor; however, a definitive diagnosis was established only after total mastectomy and immunohistochemical analysis, which confirmed STAT6 positivity consistent with SFT. Following surgery, the patient has remained recurrence-free for 5 years.

The preoperative diagnosis in our case was challenging, as imaging and core needle biopsy suggested a malignant phyllodes tumor due to rapid tumor growth. However, the definitive diagnosis of malignant SFT was made after surgical excision, which revealed a hypercellular spindle cell tumor with nuclear atypia, necrosis, and high mitotic activity. Immunohistochemical staining showed membranous and cytoplasmic positivity for CD34 and CD99, together with diffuse and strong nuclear positivity for STAT6, confirming the diagnosis. These findings are consistent with previous reports of SFT, which is histologically characterized by increased cellularity, mitotic activity, pleomorphism, necrosis, and infiltrative growth and immunohistochemically by positivity for CD34, CD99, and BCL2, with nuclear STAT6 being a highly sensitive and specific diagnostic marker for SFT. Given its rarity and overlapping features with other spindle cell tumors, such as phyllodes tumor and metaplastic carcinoma, an accurate diagnosis relies on both histopathological evaluation and STAT6 immunostaining.

Spindle cell metaplastic carcinoma is an essential diagnostic consideration in any breast spindle cell lesion, and was addressed preoperatively in the present case. It was regarded as unlikely because the core needle biopsy showed a purely mesenchymal, patternless spindle cell proliferation without an associated carcinomatous or in situ epithelial component and because the tumor cells were negative for AE1/AE3 while diffusely expressing CD34, a combination that is distinctly uncommon in metaplastic carcinoma, in which CD34 is characteristically absent.^6)^ This impression was confirmed in the resection specimen, in which the tumor was negative for AE1/AE3 and EMA throughout and no epithelial component was identified. However, it must be emphasized that cytokeratin expression in spindle cell metaplastic carcinoma is frequently focal and heterogeneous and that no single antibody labels all cases. Therefore, a panel combining broad-spectrum and high-molecular-weight cytokeratins (for example, AE1/AE3, CAM5.2, CK5/6, and 34βE12) with p63 is recommended, particularly when only limited biopsy material is available.^6)^ In retrospect, the addition of p63 and a second cytokeratin to the biopsy panel would have made the preoperative exclusion of metaplastic carcinoma more secure.

SFT is an uncommon mesenchymal neoplasm that occurs in the pleura, although it may occur throughout the body, including the head and neck region, thoracic wall, mediastinum, abdominal cavity, spinal cord, lungs, thyroid, liver, gastrointestinal tract, adrenal glands, and kidneys.^1,7–9)^ Breast involvement is exceedingly rare. Histologically, SFT typically demonstrates a patternless architecture characterized by alternating hypocellular and hypercellular areas, separated by thick bands of hyalinized collagen and thin-walled, branching, hemangiopericytoma-like vessels, referred to as staghorn vasculature.^1)^

Malignant SFTs are distinguished by hypercellularity, increased mitotic activity (usually ≥4 mitoses per 10 high-power fields), nuclear atypia, tumor necrosis, and/or infiltrative margins.^10–13)^ Immunohistochemical analysis typically reveals tumor cell positivity for CD34 (90%–95%), CD99 (70%), and BCL2, but negativity for desmin, keratin, S-100, CD117, and CD31.^10,13)^ More recently, nuclear expression of STAT6 has emerged as a highly sensitive and specific diagnostic marker for SFT.^14–16)^ In a recent comprehensive review of 231 soft tissue tumors, nuclear STAT6 staining was observed in 98% (59/60) of SFT cases, with no expression detected in other tumor types.^17)^ Malignant SFT of the breast is rare, with 7 cases reported in the English-language literature to date (**Table 1**).^4,9,18–22)^ **Table 1** summarizes the immunohistochemical and clinical data from 8 cases (including our case). Ki-67 values range from 10% to 50%, reflecting variable proliferative activity across cases.

**Table 1:** Summary of malignant SFTs of the breast

Year	Author	Reference	Age/sex	Tumor dimensions (cm)	Surgical management	Ki-67	STAT6	CD34	BCL2	CD99	Desmin	S-100	β-Catenin	p63	ER
2014	Yang	^20)^	52/F	2–3	Lumpectomy	15%	NR	+	+	+	–	–	–	–	NR
2017	Riola-Parada	^21)^	38/F	5.8	Lumpectomy	30%	NR	+	+	+	NR	NR	NR	NR	NR
2019	Jung	^4)^	75/F	4.5–5	Surgical resection		+	+	+	NR	NR	–	NR	NR	–
2019	Barco	^9)^	72/F	2–3	Wide excision	20%	+	+	+	+	–	–	–	–	–
2021	Nitta	^18)^	78/F	3.1	Modified mastectomy	50%	+	+	+	NR	NR	NR	NR	NR	NR
2023	Wang	^19)^	48/F	25	Mastectomy with sentinel lymph node biopsy	10%	+	+	+	NR	–	–	+	–	NR
2024	Ray	^22)^	52/F	10	Modified radical mastectomy	10%	+	–	+	–	–	–	–	–	NR
2025	Our case	—	67/F	16	Mastectomy	37%	+	+	+	+	–	–	+	NR	–

+, positive; –, negative; BCL2, B-cell lymphoma 2; CD, cluster of differentiation; ER, estrogen receptor; F, female; Ki-67, marker of proliferation Ki-67; M, male; NR, not reported; p63, tumor protein 63; SFTs, solitary fibrous tumors; STAT6, signal transducer and activator of transcription 6

The diagnostic value of nuclear STAT6 immunoreactivity is a direct consequence of the molecular pathogenesis of SFT. Virtually all SFTs harbor a recurrent *NAB2*-*STAT6* gene fusion generated by a paracentric inversion of chromosome 12q13, which juxtaposes *NAB2*, a transcriptional repressor of EGR1, with *STAT6.*^15)^ In the resulting chimeric protein, at least 1 repressor domain of NAB2 is replaced by the transactivation domain of STAT6, converting NAB2 from a repressor into an activator and producing constitutive activation of EGR1-mediated transcription.^15,23)^ Because the retained portion of STAT6 carries a nuclear localization signal, the fusion protein accumulates in the nucleus, and this underlies the diagnostic nuclear, as distinct from cytoplasmic, STAT6 staining, which is detected in more than 95% of SFTs and serves as a surrogate marker for essentially all fusion variants.^16,17,24)^ More than 40 fusion variants have been described, of which *NAB2*ex4-*STAT6*ex2/3 (predominating in pleuropulmonary tumors, in older patients, and in lesions with low mitotic activity) and *NAB2*ex6-*STAT6*ex16/17 (predominating in extrathoracic and meningeal tumors, in younger patients, and in more cellular lesions) are the most frequent; however, the fusion variant has not been shown to correlate consistently with recurrence or survival.^23,24)^

Genetic analysis was not performed in the present case. The tumor showed characteristic morphology together with diffuse and strong nuclear STAT6 expression, and molecular confirmation was therefore not considered necessary for the diagnosis. This accords with current practice, in which molecular testing is reserved for cases with focal, equivocal, or absent STAT6 staining, or for cases in which a STAT6-positive mimic, such as dedifferentiated liposarcoma, must be excluded.^24)^ Detection of the fusion is, in any case, technically demanding: *NAB2* and *STAT6* lie only a few hundred kilobases apart on 12q13, and the breakpoints are heterogeneous, so that break-apart fluorescence in situ hybridization is unreliable and targeted RNA sequencing or multiplex reverse transcription polymerase chain reaction is preferred.^23)^ The absence of molecular analysis is a limitation of the present report, and we are unable to specify which *NAB2*-*STAT6* variant was present in our tumor.

In our case, histopathological examination demonstrated atypical epithelioid cells in some areas within hypercellular sheets and diminished branching vasculature, together with frequent mitotic activity, tumor necrosis, and a high Ki-67 proliferation index. Positivity for CD34 and CD99, together with diffuse and strong nuclear expression of STAT6, confirmed the diagnosis of malignant SFT. However, the preoperative diagnosis did not confirm SFT, and surgery was performed because of a suspicion of a malignant phyllodes tumor. SFT is extremely rare; therefore, distinguishing it from other mesenchymal tumors based on morphological and immunohistochemical features can be difficult. However, with careful histologic examination and appropriate selection of immunohistochemical stains, SFT can be reliably differentiated from these spindle cell lesions.

A related and clinically important question is whether SFT can be diagnosed on a core needle biopsy. In principle, it can. The diagnosis rests on the recognition of a patternless proliferation of ovoid-to-spindled cells set in ropey collagen around thin-walled, branching staghorn vessels, together with diffuse and strong nuclear expression of STAT6; furthermore, STAT6 immunohistochemistry performs as well on small biopsy samples as on resection specimens.^14,16,17)^ What is required in practice is that STAT6 be included in the initial immunohistochemical panel applied to any breast spindle cell lesion in which an epithelial component is absent, rather than being reserved for the resection specimen. This was the principal missed opportunity in our case: the biopsy phenotype (CD34-positive, BCL2-positive, and cytokeratin-negative) was entirely compatible with SFT, but, in the absence of STAT6 staining, it was indistinguishable from that of a malignant phyllodes tumor, whose stromal component is also frequently positive for CD34 and BCL2.^6)^ Had STAT6 immunostaining been performed on the core biopsy specimen, a preoperative diagnosis of SFT would most likely have been reached.

Several limitations of core needle biopsy nonetheless deserve emphasis. First, the separation of SFT from malignant phyllodes tumor is intrinsically difficult on limited material, because phyllodes tumor is a biphasic lesion in which a core may sample only the overgrown stromal component, whereas entrapped native ducts within an SFT may be misinterpreted as an epithelial component of a fibroepithelial lesion. Nuclear STAT6 is the single most useful discriminator: in a series of 48 spindle cell lesions of the breast, diffuse and strong nuclear STAT6 expression was restricted to SFT, whereas all other lesions, including desmoid-type fibromatosis and spindle cell metaplastic carcinoma, were negative or showed only weak cytoplasmic staining^14)^; diffuse nuclear STAT6 staining is likewise not a feature of the stromal component of phyllodes tumor.^6)^ Second, STAT6 is highly, but not absolutely, specific: nuclear expression also occurs in dedifferentiated liposarcoma with 12q13 amplification and in *GLI1*-altered soft tissue tumors, and focal expression has been reported in other mesenchymal neoplasms; conversely, both STAT6 and CD34 expression may be attenuated or lost in dedifferentiated and high-grade SFT, so a negative result on a core taken from an anaplastic area does not exclude the diagnosis.^24)^ Third, and most relevant to a tumor such as ours, a correct core biopsy diagnosis of SFT does not, in itself, establish malignancy or predict behavior. Malignant SFT is defined by hypercellularity, at least 4 mitoses per 10 high-power fields, nuclear atypia, and necrosis, and the risk stratification models in current use require patient age, maximum tumor size, mitotic count, and the extent of necrosis to be assessed in the entire tumor.^25)^ These features are heterogeneously distributed, and a core biopsy will systematically underestimate them. For these reasons, a core needle biopsy interpretation of SFT in a rapidly enlarging breast mass should be regarded as a diagnosis to be confirmed and graded on the resection specimen. In our patient, such an interpretation would have refined the preoperative differential diagnosis and the discussion with the patient, but it would not have altered the decision to proceed with complete excision with tumor-free margins.

In the present case, although the preoperative diagnosis suggested a malignant phyllodes tumor, total mastectomy led to complete resection of a malignant SFT, resulting in favorable long-term outcomes without recurrence. Complete surgical excision with negative margins is considered the cornerstone of treatment for SFT and has been associated with reduced recurrence and improved survival.^26)^ Applying the 4-variable risk stratification model in current use,^25)^ our tumor falls into the high-risk group: age ≥55 years (1 point), maximum size ≥15 cm (3 points), and a mitotic count of 32 per 50 high-power fields, corresponding to approximately 6.4 per 10 high-power fields (2 points), which amount to 6 points before the extent of necrosis is considered. The necrosis, which was macroscopically extensive in the central portion of the tumor, was not formally quantified; scoring it as 10% or more would give a total of 7 points, so the tumor is classified as high risk irrespective of how necrosis is scored. Nevertheless, the patient has remained free of recurrence and metastasis for 5 years after complete excision. This discrepancy suggests that risk models derived largely from pleuropulmonary and deep soft tissue SFTs may not be directly transferable to the SFTs of the breast, and it supports close long-term follow-up rather than adjuvant therapy after complete resection. However, due to the rarity of malignant SFT of the breast, standardized systemic or targeted therapies have not been established, particularly in cases of advanced or metastatic disease.^27–32)^ While some extrapolated data suggest a generally favorable prognosis for malignant SFT compared to other soft tissue sarcomas, the evidence remains limited. Reports to date, including those of malignant SFTs of the breast, have rarely documented recurrence or metastasis, suggesting that complete resection may suffice in localized disease. Nevertheless, diagnostic uncertainty—particularly in distinguishing SFT from other spindle cell lesions—underscores the need for accurate histopathologic assessment supported by STAT6 immunohistochemistry to inform appropriate surgical and follow-up strategies.

## CONCLUSIONS

Malignant SFT of the breast is an extremely rare entity that poses diagnostic challenges due to its overlapping features with other spindle cell tumors. Our case highlights the importance of considering SFT in the differential diagnosis of rapidly growing breast masses and demonstrates that complete surgical excision can result in favorable outcomes. Accurate histopathological evaluation, particularly with STAT6 immunostaining, is essential for definitive diagnosis and appropriate management. STAT6 should therefore be included in the immunohistochemical panel applied to core needle biopsy specimens of breast spindle cell lesions that lack an epithelial component, although the assessment of malignancy and risk still requires the resection specimen.

## DECLARATIONS

### Availability of data and materials

All data supporting the conclusions of this article are included within the article.
